# Automated quantification of avian influenza virus antigen in different organs

**DOI:** 10.1038/s41598-024-59239-5

**Published:** 2024-04-16

**Authors:** Maria Landmann, David Scheibner, Marcel Gischke, Elsayed M. Abdelwhab, Reiner Ulrich

**Affiliations:** 1 Institute of Veterinary Pathology, Leipzig University, Leipzig, Germany; 2 Institute of Molecular Virology and Cell Biology, Friedrich-Loeffler-Institut, Greifswald-Insel Riems, Germany

**Keywords:** Experimental models of disease, Image processing

## Abstract

As immunohistochemistry is valuable for determining tissue and cell tropism of avian influenza viruses (AIV), but time-consuming, an artificial intelligence-based workflow was developed to automate the AIV antigen quantification. Organ samples from experimental AIV infections including brain, heart, lung and spleen on one slide, and liver and kidney on another slide were stained for influenza A-matrixprotein and analyzed with QuPath: Random trees algorithms were trained to identify the organs on each slide, followed by threshold-based quantification of the immunoreactive area. The algorithms were trained and tested on two different slide sets, then retrained on both and validated on a third set. Except for the kidney, the best algorithms for organ selection correctly identified the largest proportion of the organ area. For most organs, the immunoreactive area assessed following organ selection was significantly and positively correlated to a manually assessed semiquantitative score. In the validation set, intravenously infected chickens showed a generally higher percentage of immunoreactive area than chickens infected oculonasally. Variability between the slide sets and a similar tissue texture of some organs limited the ability of the algorithms to select certain organs. Generally, suitable correlations of the immunoreactivity data results were achieved, facilitating high-throughput analysis of AIV tissue tropism.

## Introduction

Avian influenza viruses (AIV) infect a wide range of bird species and other animals^1,2^. Influenza viruses belong to the family *Orthomyxoviridae*. Depending on the surface antigens hemagglutinin (HA) and neuraminidase (NA), different HxNy-subtypes are defined^3^. AIV in birds can be further divided into highly pathogenic AIV (HPAIV) and low pathogenic AIV (LPAIV). While HPAIV infection causes severe systemic disease, LPAIV infection often causes no or only mild clinical disease, usually limited to the respiratory and/or gastrointestinal tract in chickens, respectively^4^. Influenza viruses have a high variability due to multiple mechanisms such as mutations (antigenic drift), and reassortment, i.e., an exchange of whole gene segments (antigenic shift)^1,5^. Due to this variability and many other factors, such as host species or host age, the tissue tropism and pathology of different AIV subtypes in different infected species are also highly variable^6–8^ and HPAIV infection is often associated with necrotizing lesions and lymphoid depletion in many organs^4,9^. Especially in the case of HPAIV infections in chickens, a considerable amount of viral antigen is often also present in endothelial cells^10^, contributing to the systemic spread of the virus^4^.

In pathology, the viral antigen distribution is often assessed by immunohistochemical staining of the antigen followed by microscopic examination. This method allows for a qualitative assessment of the tissue tropism with identification of the different affected cell types in various organs (e.g., parenchymal, endothelial or immune cells), the preference for a specific localization inside the cells (e.g., nuclear or cytoplasmic) in these organs and the examination of their association with lesions such as necrosis^11^. Also, immunohistochemistry detects viral antigens (i.e., proteins), thus enabling conclusions about the active virus replication in specific cells. In addition, RT-PCR data are influenced by viruses circulating in the blood that do not represent organ infection per se. Immunohistochemistry is often used as a valuable supplementation for these analyses to further elucidate AIV tissue tropism and in vivo pathogenesis. Furthermore, a semiquantitative score is often applied to facilitate a comparison between different viruses and animals^8^. Both of these methods are relatively time-consuming and thus often only a small number of animals are examined. This in turn limits the statistical analysis of the collected data.

In contrast, digital quantification of AIV antigen can enable automated, high-throughput analysis of tissue tropism. The advantages of automated analysis, in addition to reduced workload, include consistency of assessment and the ability to measure and/or count many structures not only in representatively chosen areas of the slides but on whole slide images and for a large number of samples^12,13^. However, there are limitations to computer-based analysis regarding the complexity of the task as well as the variability and quality of the slides analyzed^12–14^.

QuPath is an open-source software specially designed for whole slide image analysis, which provides a large number of tools for slide assessment and has many options for integrating different steps of analysis in one workflow, thus enabling largely automated image analysis^15^.

This study aimed to create and validate a method for automated analysis of tissue samples, detecting and quantifying influenza virus antigen, enable a high-throughput evaluation of tissue samples, and reduce the associated workload for the examining pathologists. Automated organ selection was established as a first step since such studies often strive to examine many different tissues and therefore, a histopathologic slide frequently contains samples of multiple different organs. As often only a subset of organs is subjected to and fit for specific analyses, the presence of additional tissue samples on one slide was used in this study to assess possible limitations of the automated organ selection. In the second step, the immunoreactive area was quantified by applying threshold-based pixel classifiers in the respective organ regions selected in the first step.

## Material and methods

### AIV infection experiments

For the training and validation of the algorithms and the threshold-based antigen quantification, organ samples from multiple AIV infection studies (studies 1–3) conducted at the Friedrich-Loeffler-Institut (FLI; Greifswald-Insel Riems, Germany) were used. In these studies, approximately 6-week-old white leghorn chickens from Lohmann Animal Health (Cuxhaven, Germany) were inoculated with 12 different AIVs either oculonasally (studies 1, 2, 3) with 0.2 ml containing 10^5^ plaque-forming units (PFU) per bird, or intravenously (study 3) with 0.1 ml of 1:10 diluted allantoic fluid^16^, according to WOAH/OIE recommendations^17^, and died or were euthanized after inhalation of Isoflurane® (CP-Pharma, Germany) at 1–4 days post inoculation (dpi). Seven non-infected chickens were used as a negative control group. All experiments were approved by the State Office of Agriculture, Food Safety and Fishery in Mecklenburg-Western Pomerania, Germany (LALLF M-V, registration numbers LALLF MV 7221.3-1-060/17 and 7221.3–1.1-051-12) and performed in accordance with all relevant guidelines and regulations, as well as in compliance with the ARRIVE guidelines.

### Histology and immunohistochemistry

Animals were necropsied under biosafety level-3 (BSL-3) conditions following standard procedures. Organ samples of the brain, heart, lung, spleen, liver, and kidney, as well as multiple other organs (skin, nasal cavity, trachea, thymus, glandular stomach, gizzard, duodenum, jejunum, caecum, pancreas, and bursa fabricii), were taken, fixed in 4% neutral-buffered formaldehyde for > 7 days, processed and embedded in paraffin wax. For the study groups, one paraffin block (block A) contained brain, heart, lung, and spleen and another block (block B) contained liver, kidney, glandular stomach, gizzard, thymus, and trachea. 4–5 µm microtome slices were mounted on glass slides. Immunohistochemical examination was conducted with the avidin–biotin-peroxidase complex (ABC) method (Vectastain PK-6100; Vector Laboratories, Newark, CA, USA) with citric buffer pretreatment (pH 6.0), a primary monoclonal mouse antibody targeting an epitope of the influenza A matrixprotein (ATCC clone HB-64, 1:100), a secondary biotinylated goat-anti-mouse IgG (BA-9200, Vector Laboratories, Newark, USA, 1:200), 3-amino-9-ethylcarbazol (AEC) as chromogen (Agilent Technologies, Santa Clara, CA, USA and Nichirei Biosciences Inc., Tokyo, Japan), and hematoxylin counterstain as done before^18,19^. Controls included validated positive and negative archival tissues^19^, as well as replacement of the primary antibody with an anti-isotype IgG antibody.

### Setup for image analysis

Before scanning, the slides were checked for artifacts such as dust, contaminants, severe tissue folds, or air bubbles. If present, these were minimized as much as possible, with the aim of keeping artifacts to less than 0.5% of the organ area. Slides were scanned using the AxioScan 7 with ZEN blue software (Carl Zeiss Microscopy GmbH, Jena, Germany), a 20 × objective with a numeric aperture of 0.45 and a pixel size of the scanned slides of 0.1725 × 0.1725 µm (detailed settings see Supplementary Table S1) and stored via ZEN data storage (Carl Zeiss Microscopy GmbH, Jena, Germany). Image analysis was conducted with QuPath v0.2.3^15^ on desktop computers with at least 16 GB RAM and a 3.30 GHz processor and monitors with at least 59.8 Hz/1920 × 1080.

First, one training set consisting of 21 animals and one test set consisting of 12 animals was selected from the chickens of studies 1 and 2. The 15 chickens of study 3 were used as a validation set, as they were independent of the chickens of studies 1 and 2.

### Automated selection of organs

In short, two separate classifiers were trained for the automated organ selection on the immunohistochemical tissue sections using the “object classification” tools: one for block A/slide A to select the brain, heart, lung, and spleen and one for block B/slide B to select the liver and kidney among other tissue samples, which were present on slide B, but not used for quantitative analysis. For a short overview of the training and evaluation of the classifiers, see Supplementary Fig. S1, for a detailed workflow see Supplementary Figs. S2–S6.

For training of the classifiers, representative regions of the different organs were manually annotated on the slides of the training set and sometimes of the test set. Classes used for the annotations were *brain*, *heart*, *lung*, *spleen*, and *background*, including clear space and artifacts, for slide A, and *liver*, *kidney*, and *background*, including clear space, artifacts, and other organs present on the slide, for slide B. Furthermore, for training and evaluation, slides of the training set and test set were divided into tiles of 1000 × 1000 µm using the “Create tiles” tool. Of these created tiles, one tile for each organ and one tile for the background were selected randomly using Microsoft Excel (Version 2108, Microsoft 365) and annotated at 6× display magnification and the rest of the tiles were discarded (Supplementary Fig. S2). This was done for 12 randomly selected animals of the training set and all 12 animals of the test set. For 7 animals of the validation set, one 1000 × 1000 µm tile was manually annotated for evaluation, likewise. Additionally, on all slides of all sets a rough outline of each organ was manually annotated at 1–2× display magnification for evaluation purposes.

Stain vectors were set via the “Estimate stain vectors” tool based on one slide from study 1, and stain vectors were then transferred to all other slides (Supplementary Table S2). Subsequently, the “SLIC superpixel segmentation” tool was used on the whole slides to create multiple small sub-regions consisting of similar pixels. Intensity features were then calculated for each superpixel (Supplementary Tables S3 and S4).

Using the annotations of representative regions and tiles in the training set, a set of initial classifiers was trained via the “Train object classifier” tool. All trained classifiers were random trees classifiers and default settings for features were used, as they were found to work best in visual pre-evaluation. These initial classifiers were then applied to all slides of the training set as well as the test set, each consisting of animals from studies 1 and 2, to assess performance on slides known to the classifier as well as on slides unknown, but originating from the same studies. This resulted in one class being assigned to each superpixel (Supplementary Fig. S3).

The classification of the superpixels was then transformed into larger annotations for each class using the “Tile classifications to annotations” tool. Annotations and holes in annotations smaller than 1,000,000 µm^2^ were removed via the “Remove fragments & holes” tool to exclude smaller, falsely classified regions, resulting in one large, cohesive, annotated region for each organ sample (Supplementary Fig. S3).

Results of the classification of the slides were then evaluated visually as well as using the 1000 × 1000 µm tiles and the rough outlines of the organs to allow for assessment at different levels of detail. For the tile evaluation, labeled images downsampled to a pixel size of 1 × 1 µm were extracted for each tile from the automated as well as the manually annotated slides. These labeled images were compared using the MorphoLibJ plugin^20^ for Fiji/ImageJ^21^ (ImageJ version 1.53q) to calculate the Jaccard similarity index (Supplementary Fig. S4). For further evaluation of the whole slides, the percentage of correctly classified tissue (e.g., brain correctly classified as *brain*) or falsely classified tissue (e.g., brain falsely classified as *heart*, *lung*, *spleen,* and/or *background*) was calculated for each rough organ outline (Supplementary Fig. S5).

The initial classifiers trained with the training set were evaluated as described above and the best-performing classifiers were identified. These classifiers were then re-trained with the manual annotations from the test set in addition to those from the training set to further improve performance. These refined classifiers were then applied to the validation set, consisting of animals from study 3, as well as to the training set and test set, and evaluated as described above. Therefore, the performance on slides unknown to the classifier and originating from a study independent of the studies used in the training set and the test set was assessed.

### Threshold-based quantification of immunoreactive area

The immunoreactive area was then measured using threshold-based pixel classifiers via the “create thresholder” tool in the selected organ regions (Supplementary Fig. S6). Organ-specific suitable thresholds for the AEC signal (immunoreactive area) were defined by visually assessing selected representative slides from at least 7 animals of the training set as well as slides from the 7 non-infected control animals for each organ (Table 1). A moderate resolution (1.38 µm/pixel) was chosen for the classification of the immunoreactive area. Those organ-specific thresholds were then applied to the respective selected organ region on the slides of the training set, test set, and validation set. The immunoreactive area was then transformed into an annotation with a minimum object and hole size of 3 µm^2^. The immunoreactive area and immunonegative area were then measured and the percentage of the immunoreactive area was calculated per organ. This detection of the immunoreactive area was done for the manually selected regions as well as the regions selected by the established organ classifiers.

**Table 1:** Threshold for detection of immunoreactive area.

Organ	Threshold channel “DAB”*
Brain	0.09
Heart	0.1
Lung	0.13
Spleen	0.11
Liver	0.18
Kidney	0.18

*Names are pre-set by QuPath, channel “DAB” was adapted for AEC staining; further thresholder settings: resolution = moderate (1.38 µm/px), Smoothing sigma = 0.

As the immunohistochemical slides of the liver and kidney showed variable and often pronounced false positive immunoreactivity in peripheral regions, the margins of the annotations for the liver and kidney were excluded before the threshold-based antigen quantification. Margin width was chosen based on the estimation of the peripheral false positive area of the training set and the non-infected control animals, which resulted in a circumferential subtraction of 400 µm for the liver and 500 µm for the kidney. This was applied to all slide sets using the “expand annotation” tool.

### Creation of a coherent workflow

All the steps necessary for pre-processing, organ selection, post-processing, and threshold-based quantification of the immunoreactive area were compiled into one coherent script to enable unsupervised batch-processing of multiple slides with QuPath.

### Correlation of quantitative and semiquantitative analysis data

A semiquantitative scoring of parenchymal and endothelial antigen was conducted^8^. In short, the distribution of viral antigen was scored as follows: for parenchymal cells 0 = no, 1 = focal to oligofocal, 2 = multifocal, 3 = coalescing to diffuse antigen and for endothelial cells 0 = no antigen, 1 = antigen in single blood vessels, 2 = antigen in multiple blood vessels, 3 = diffuse immunoreactivity. For this study, the score was used as a parenchymal antigen score only or as the sum of the scores for parenchymal and endothelial antigen. Spearman’s correlation analysis was done with GraphPad Prism (Prism 8 for Windows, version 8.4.3, GraphPad Software, San Diego, CA, USA) to compare the percentage of immunoreactive area with the semiquantitative score.

In cases where the classifiers falsely selected no organ area when the respective organ was present on the slide, or selected organ area on a slide when the respective organ was not present, the pair of values was excluded.

### Cutoff value for immunoreactive and immunonegative organs, sensitivity, and specificity

Unspecific staining and artifacts could not be fully excluded by the pixel classifier based on an AEC threshold alone without losing the majority of the true immunoreactive signal of the viral antigen. Therefore, an additional cutoff value was determined for the percentage of the threshold-based area to discern positive (i.e., displaying viral antigen) and negative organs (i.e., displaying no true viral antigen, but sometimes unspecific staining or artifacts). For this, the semiquantitative score described above^8^ was used as a ground truth as follows: If the sum of the parenchymal score and the endothelial score was 0, the organ was defined as negative ground truth and otherwise as positive ground truth. For the establishment of the cutoff value, the percentage of the immunoreactive area measured inside the manual organ selections was compared to this ground truth as follows: For the training set and the negative control animals, different cutoff values and the associated number of false positive and false negative samples for each organ were calculated using Microsoft Excel (Version 2108, Microsoft 365). One appropriate cutoff value per organ was selected accordingly and applied to the other data sets, including the slides with automated organ selection. As described for the correlation analysis, the pair of values was excluded if no organ area was selected for a present organ or organ area was falsely selected without the respective organ being present on the slide. The sensitivity and specificity were then calculated for organs of the training set, test set, and validation set, likewise.

### Comparison of immunoreactive area for the validation set

For the 15 chickens of the validation set infected with the same H5N1 HPAIV^16^, the percentage of the immunoreactive area was compared between the animals with intravenous and oculonasal inoculation routes at 2 dpi using Mann–Whitney U tests and between the different organs within one inoculation group using Friedman tests followed by Dunn’s post-hoc-tests with GraphPad Prism (Prism 8 for Windows, version 8.4.3, GraphPad Software, San Diego, CA, USA). Due to missing values, the kidney was fully excluded from the automated organ selection and subsequently only animals with a full set of measurements from all organs were included in the statistical analysis.

### RT-qPCR

For the animals of study 1, samples of brain, heart, lung, spleen, liver, and kidney (n = 94) were analyzed by reverse transcription-quantitative polymerase chain reaction (RT-qPCR), as described before^22^. Samples were homogenized and RNA extraction was done with NucleoMag VET kit (Macherey–Nagel GmbH, Germany) and KingFisher (Thermo Fisher Scientific, USA), following the manufacturer’s instructions. RT-qPCR was done using 1-Step RT-qPCR ToughMix for M1 (Quantabio, MA, USA). The relative virus amount (equivalent log10 PFU/ml) was calculated using standard curves. A Spearman’s correlation analysis and Pearson’s correlation analysis was performed to correlate the RT-qPCR data with the semiquantitative score and the percentage of the immunoreactive area, respectively, using GraphPad Prism (Prism 8 for Windows, version 8.4.3, GraphPad Software, San Diego, CA, USA).

## Results

### Evaluation of automated organ selection

**Figure 1:**
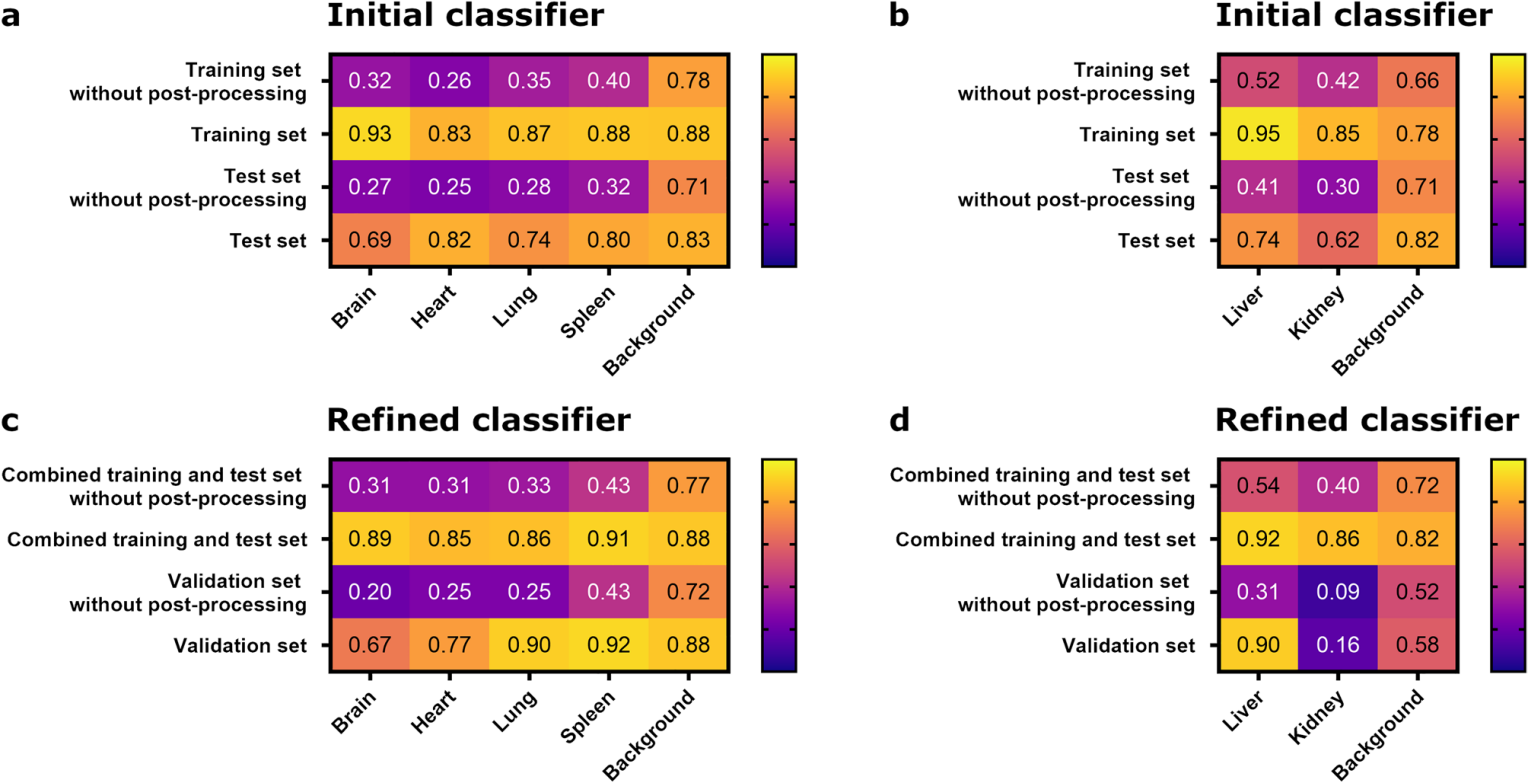
Evaluation of organ identification using automated random trees classifiers compared to manual annotation. Mean Jaccard similarity index (0 = no similarity, 1 = full similarity) for different classes assigned by the initial (**a**, **b**) and refined (**c**, **d**) classifier algorithms compared to manual annotation in randomly selected 1000 × 1000 µm tiles. This was done before and after post-processing removal of annotations and holes in annotations smaller than 1,000,000 µm^2^. (**a**, **c**) Brain, heart, lung, spleen and background; (**b**, **d**) liver, kidney and background. Number of randomly selected tiles (note that not all organs were present on all tiles): (**a**) training set n = 59, test set n = 59; (**b**) training set n = 36, test set n = 35; (**c**) combined training and test set n = 118, validation set n = 32; (**d**) combined training and test set n = 71, validation set n = 21

Classifiers for automated organ selection were trained, evaluated, refined, and applied. For the best-performing classifiers, the Jaccard similarity index was calculated for selected tiles, comparing the automated organ selection to a detailed manual selection. Tiles of the training set and test set were used for evaluation of the initial classifiers and tiles of the combined training and test set and the validation set were used for evaluation of the refined classifiers. For each classifier, a post-processing exclusion of regions smaller than 1,000,000 µm^2^ was conducted and compared to the same regions without this optimization. In most cases, the mean Jaccard indices were considerably higher for the post-processed organ selections than for the selections without this processing step (Fig. 1). Generally, the mean Jaccard indices of the slide sets used for the training of the initial classifiers, i.e., the training set, and the refined classifiers, i.e., the combined training and test set, were higher than those for the slide sets, which were not used for training, i.e., test set and validation set, respectively. With exception of the kidney and the background in the validation set, mean Jaccard indices for post-processed organ selections by the best classifiers were above 0.6 throughout all slide sets (Fig. 1).

**Figure 2:**
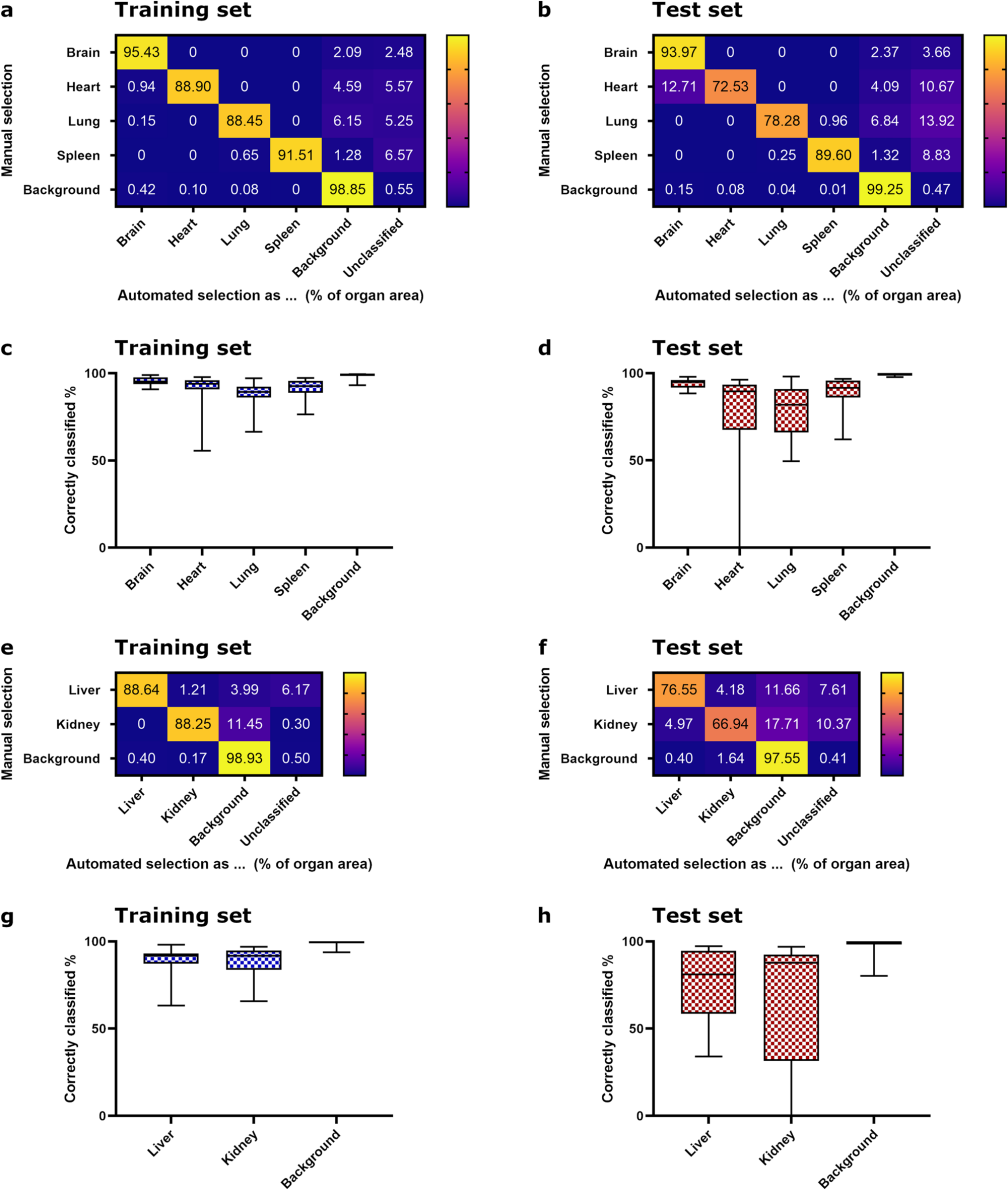
Evaluation of initial classifiers compared to manual annotation for whole slide images. (**a**, **b**, **e**, **f**) Mean percentages of classes assigned by the initial classifier algorithms compared to a manual ground truth annotation of the organs for brain, heart, lung, spleen and background and liver, kidney and background, respectively. E.g., inside the manually annotated ground truth area of the brain of the training set, on average, 95.43% of the area were classified correctly as *brain*, 2.09% were classified as *background* and 2.48% were unclassified due to removal of small fragments during post-processing. (**a**, **e)** Training set, (**b**, **f**) test set. (**c**, **d**, **g**, **h**) Box plots with minimum and maximum of correctly classified area by the initial classifier algorithms for each organ per set. Median is depicted as horizontal line. (**c**, **g**) Training set, (**d**, **h**) test set. Sample size: training set n = 21 (brain, lung, liver, kidney, background) or n = 20 (heart, spleen); test set n = 12 (brain, heart, lung, kidney, background) or n = 11 (spleen, liver).

**Figure 3:**
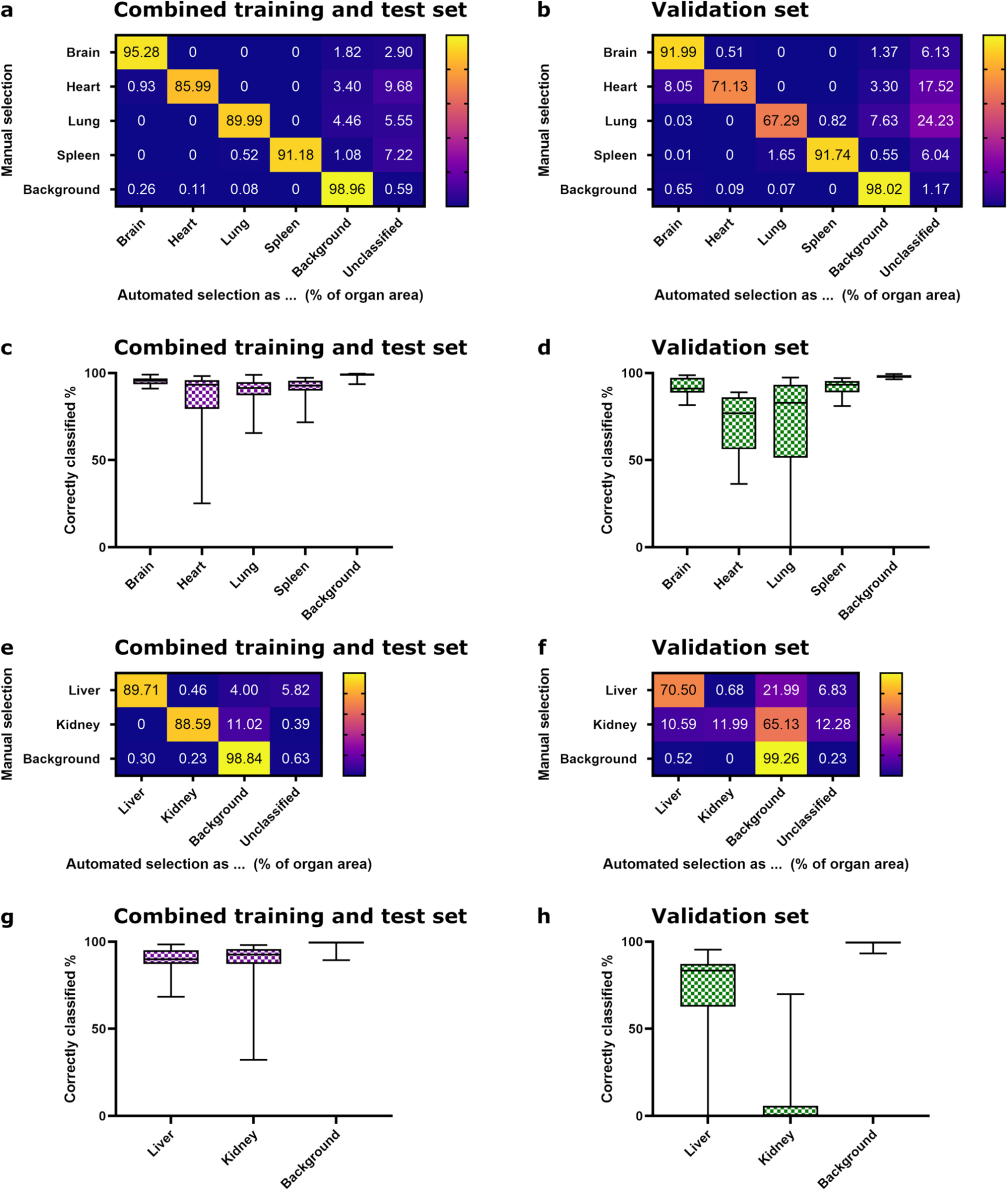
Evaluation of refined classifiers compared to manual annotation for whole slide images. (**a**, **b**, **e**, **f**) Mean percentages of classes assigned by the refined classifier algorithms compared to a manual ground truth annotation of the organs for brain, heart, lung, spleen and background and liver, kidney and background, respectively. E.g., inside the manually annotated ground truth area of the brain of the validation set, on average, 91.99% of the brain area were classified correctly as *brain*, 0.51% were classified as *heart*, 1.37% as *background* and 6.13% were unclassified due to removal of small fragments during post-processing. (**a, e**) Combined training and test set, (**b**, **f**) validation set. (**c**, **d**, **g**, **h**) Box plots with minimum and maximum of correctly classified area by the refined classifier algorithms for each organ per set. Median is depicted as horizontal line. (**c**, **g**) Combined training and test set, (**d**, **h**): validation set. Sample size: combined training and test set n = 33 (brain, lung, kidney, background), n = 32 (heart, liver) or n = 31 (spleen); validation set n = 15 (heart, lung, liver, kidney, background), n = 14 (spleen) or n = 13 (brain).

Furthermore, for the best-performing classifiers, the percentage of correctly classified tissue was calculated for the whole slides after post-processing utilizing the rough, manual outline. Similar to the Jaccard index, the mean correctly selected percentage of each organ was generally higher for the slide sets used for training the classifiers than for the slide sets not used for training the initial and the refined classifiers, likewise (Figs. 2, 3). The mean correctly selected percentage was mostly above 65% except for the kidney of the validation set (Figs. 2, 3). In case of the kidney, only 11.99% were classified correctly (Fig. 3 f). Most of the misclassified organ area was classified as background or was left unclassified due to the removal of small fragments during post-processing. The mean percentage of the area of one organ, which was misclassified as another organ, was less than 5% per slide set for most organs and sets, except for the kidney in the validation set as selected by the refined classifier and the heart in the test set and validation set as selected by the initial and refined classifier, respectively (Figs. 2 b, 3b, f). In the case of the kidney, 10.59% were falsely classified as *liver* and 65.13% were falsely classified as *background* (Fig. 3f), and in the case of the heart, 12.71% for the test set and 8.05% for the validation set were falsely classified as *brain* (Figs. 2b, 3b).

### Evaluation of quantification of immunoreactive area

**Figure 4:**
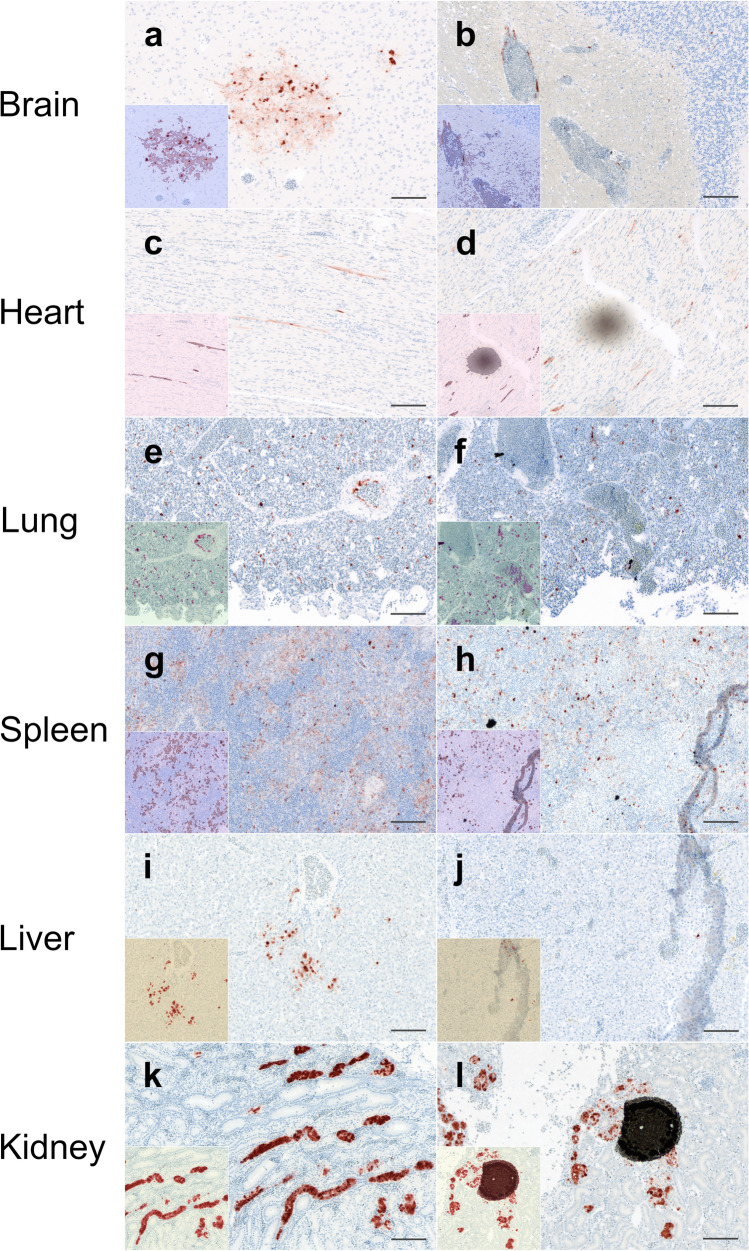
Results and limitations of threshold-based quantitative analysis. Representative images from scanned slides of the training and test set before (large images) and after (insets) automated analysis with QuPath. The left column (**a**, **c**, **e**, **g**, **i**, **k**) illustrates good performance of automated threshold-based analysis, while the right column (**b**, **d**, **f**, **h**, **j**, **l**) illustrates suboptimal false positive detection due to common pitfalls such as incompletely blocked endogenous peroxidase activity in erythrocytes (**b**, **f**), tissue folds (**h**, **j**), dust (**d**) and other foreign particles (**f**, **h**, **l**). The insets display a semi-transparent, pseudo-colored overlay that highlights the area of immunoreactivity that was automatically detected: (**a**, **b**) brain: dark violet = positive area, blue = negative area; (**c**, **d**) heart: purple = positive area, pale pink = negative area; (**e**, **f**) lung: purple = positive area, green = negative area; (**g**, **h**) spleen: purple = positive area, pale violet = negative area; (**i**, **j**) liver: dark red = positive area, yellow–brown = negative area; (**k**, **l**) kidney: light red = positive area, pale yellow = negative area. Influenza matrixprotein with hematoxylin counterstain, bars = 100 µm.

**Figure 5:**
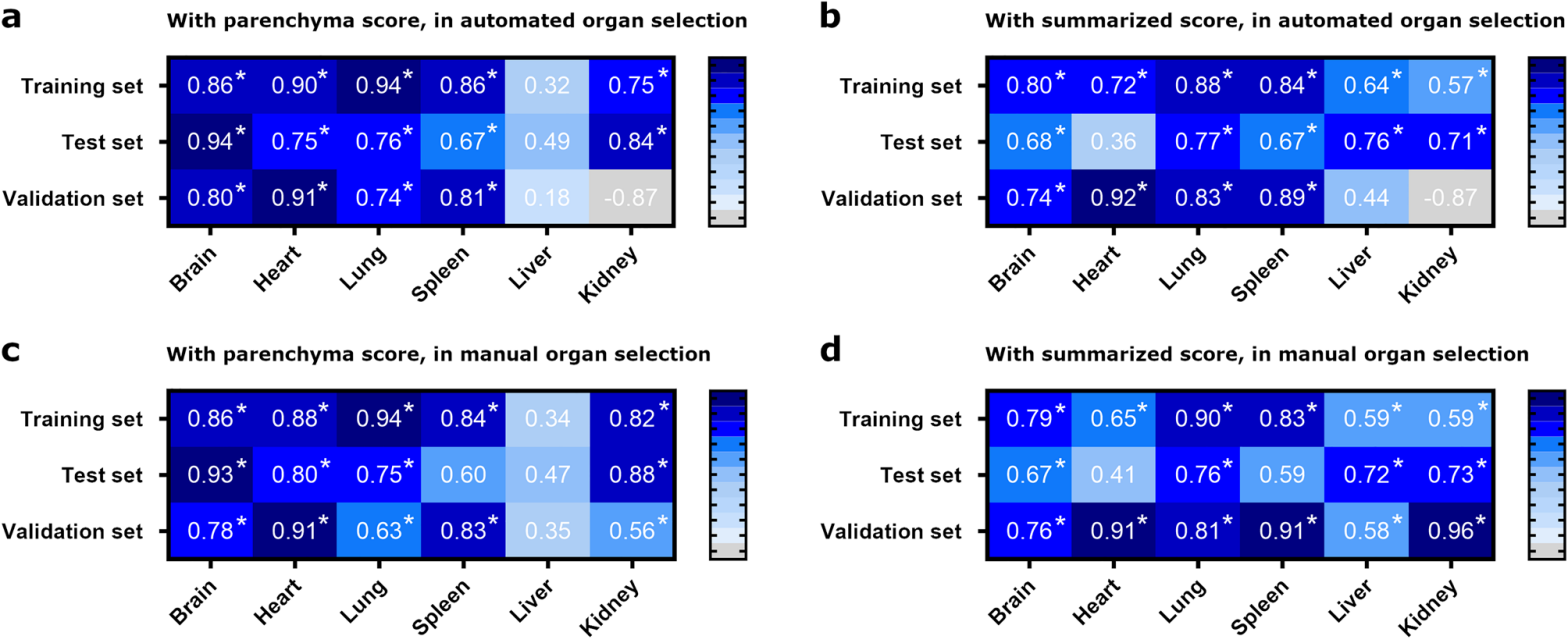
Spearman correlation analysis of the percentage of immunoreactive area and a semiquantitative score. Spearman correlation coefficients for the percentage of threshold-based immunoreactive area with manually assessed, semiquantitative scores for parenchymal and endothelial antigen in classifier-based and manual organ selection. (**a**) Correlation of area measured in automated organ selection with refined classifiers with semiquantitative score of parenchymal antigen, (**b**) correlation of area measured in automated organ selection with refined classifiers with sum of semiquantitative parenchymal and endothelial organ scores, (**c**) correlation of area measured in manual organ selection with semiquantitative score of parenchymal antigen, (**d**) correlation of area measured in manual organ selection with sum of semiquantitative parenchymal and endothelial organ scores. *: p ≤ 0.05. Sample size: (**a**, **b**) training set n = 21 (brain, lung, liver, kidney) or n = 20 (heart, spleen), test set n = 12 (brain, heart, lung, kidney) or n = 11 (spleen, liver), validation set n = 15 (heart), n = 14 (spleen), n = 13 (brain, lung, liver) or n = 3 (kidney); (**c**, **d**) training set n = 21 (brain, lung, liver, kidney) or n = 20 (heart, spleen), test set n = 12 (brain, heart, lung, kidney) or n = 11 (spleen, liver), validation set n = 15 (heart, lung, liver, kidney), n = 14 (spleen) or n = 13 (brain).

The automated organ selection of the best refined classifiers was used as a basis for the threshold-based quantification of the immunoreactive area (Fig. 4). For evaluation, the same classifiers were additionally applied to the manual organ selections. The results of both analyses were compared to the semiquantitative score for parenchymal antigen only and the sum of the scores for parenchymal and endothelial antigen, respectively. Throughout all analyses, the percentage of the immunoreactive area showed a positive and often significant (p ≤ 0.05) correlation with the semiquantitative scores, except for the kidney in the automated organ selection, which showed a negative correlation (Fig. 5).

### Comparison of infection routes for the validation set

**Figure 6:**
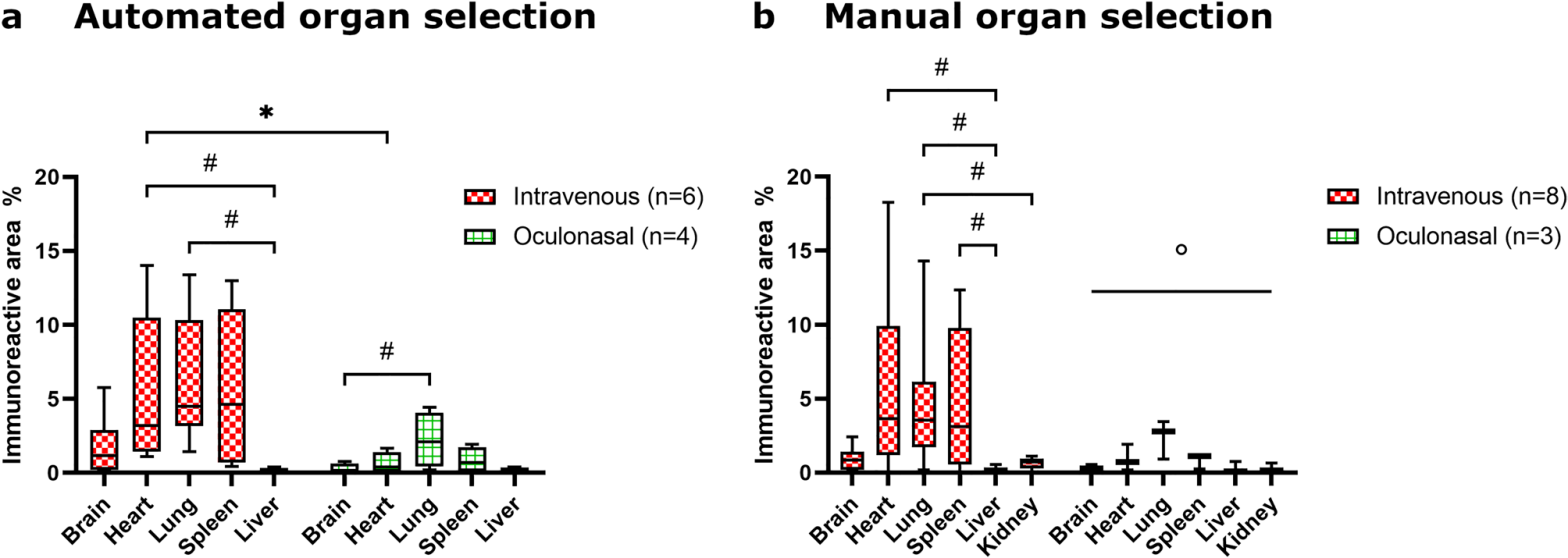
Comparison of percentage of immunoreactive area for H5N1 infected chickens. Box plots with minimum and maximum of percentage of immunoreactive area for chickens infected intravenously and oculonasally in classifier-based, automated (**a**) and manual organ selection (**b**). Median is depicted as horizontal line. Between groups, organs were compared using Mann–Whitney test, inside one group, organs were compared using Friedman test with Dunn’s post-hoc test. */#/°: p ≤ 0.05 (*: Mann–Whitney-Test; #: Friedman-Test with Dunn’s post-hoc-Test; °: Friedman-Test p ≤ 0.05 and Dunn’s post-hoc-Test > 0.05). Sample size: automated organ selection intravenous n = 6, oculonasal n = 4, manual organ selection intravenous n = 8, oculonasal n = 3.

The immunoreactive area was compared for the organs of the chickens of the validation set at 2 dpi, which were infected with the same H5N1 virus via IV or ON inoculation route. In general, the immunoreactive area measured in both manually and automatically selected organs exhibited a similar pattern across different organs and infection routes: The IV-infected animals had a larger percentage of immunoreactive area than the ON-infected animals and brain, liver, and kidney had a smaller percentage of immunoreactive area than heart, lung, and spleen. For the automated organ selections, the immunoreactive area of the heart was significantly smaller in the ON-infected animals than in the IV-infected animals (Mann–Whitney U test, p ≤ 0.05). In the IV-infected animals, the immunoreactive area in the liver was significantly smaller than in the lung and heart. For the ON-infected animals, the immunoreactive area in the brain was significantly smaller than in the lung (p ≤ 0.05, Friedman test with Dunn’s post-hoc-tests) (Fig. 6a). Considering the manual organ selection, in the IV-infected animals the liver had a significantly smaller immunoreactive area compared to the heart, lung, and spleen. Additionally, the kidney had a significantly smaller immunoreactive area than the lung (p ≤ 0.05, Friedman tests with Dunn’s post-hoc-tests). The immunoreactive area of the six organs of the ON-infected animals differed significantly with the Friedman test (p ≤ 0.05), but no significant difference between any pair of two organs was detected with Dunn’s post-hoc-test (Fig. 6b).

### Cutoff value for organ positivity

To allow for an additional quick assessment of the overall immunoreactivity of an organ and to account for occasional false positive signal due to unspecific staining and artifacts, a cutoff value was determined for the whole organ. This was based on the measured percentage of immunoreactive area to distinguish “positive” from “negative” organs. Appropriate cutoff values were determined using the negative control animals and the training set with the manual organ selection. These cutoff values varied between the organs and ranged from ≥ 0.06 to ≥ 0.13% of the immunoreactive area. The selected cutoff values were then applied to the training set, test set, and validation set both with manual and automated organ selection and were evaluated using a semiquantitative score as a ground truth measurement for positivity and negativity (Tables 2 and 3). Sensitivity, specificity, and accuracy for the cutoff value were often suitable, but overall showed high variability.

**Table 2:** Evaluation of cutoff value for immunoreactive and immunonegative organs—manual organ selection.

Manual organ selection	n	fp	fn	tp	tn	gtp	gtn	Sensitivity	Specificity	Accuracy
Brain cutoff ≥ 0.09%										
Negative control	7	1	0	0	6	0	7	n. a.	85.7%	85.7%
Training set	21	0	3	18	0	21	0	85.7%	n. a.	85.7%
Test set	12	1	1	10	0	11	1	90.9%	0.0%	83.3%
Validation set	13	0	2	11	0	13	0	84.6%	n. a	84.6%
Heart cutoff ≥ 0.13%										
Negative control	7	0	0	0	7	0	7	n. a.	100.0%	100.0%
Training set	20	0	3	17	0	20	0	85.0%	n. a.	85.0%
Test set	12	0	1	10	1	11	1	90.9%	100.0%	91.7%
Validation set	15	0	2	11	2	13	2	84.6%	100.0%	86.7%
Lung cutoff ≥ 0.09%										
Negative control	7	1	0	0	6	0	7	n. a.	85.7%	85.7%
Training set	21	0	3	15	3	18	3	83.3%	100.0%	85.7%
Test set	12	2	0	6	4	6	6	100.0%	66.7%	83.3%
Validation set	15	1	1	13	0	14	1	92.9%	0.0%	86.7%
Spleen cutoff ≥ 0.06%										
Negative control	7	1	0	0	6	0	7	n. a.	85.7%	85.7%
Training set	20	1	6	13	0	19	1	68.4%	0.0%	65.0%
Test set	11	1	2	5	3	7	4	71.4%	75.0%	72.7%
Validation set	14	2	1	10	1	11	3	90.9%	33.3%	78.6%
Liver cutoff ≥ 0.12%										
Negative control	7	2	0	0	5	0	7	n. a.	71.4%	71.4%
Training set	21	1	8	9	3	17	4	52.9%	75.0%	57.1%
Test set	11	1	4	3	3	7	4	42.9%	75.0%	54.5%
Validation set	15	1	6	5	3	11	4	45.5%	75.0%	53.3%
Kidney cutoff ≥ 0.06%										
Negative control	7	0	0	0	7	0	7	n. a.	100.0%	100.0%
Training set	21	0	1	20	0	21	0	95.2%	n. a.	95.2%
Test set	12	0	1	10	1	11	1	90.9%	100.0%	91.7%
Validation set	15	0	3	11	1	14	1	78.6%	100.0%	80.0%

% = percentage of immunoreactive area; n: animal number; fp: false positive; fn: false negative; tp: true positive; tn: true negative; gtp: ground truth positive (= semiquantitative score > 0); gtn: ground truth negative (= semiquantitative score = 0); sensitivity = tp/gtp; specificity = tn/gtn; accuracy = (tp + tn)/(tp + tn + fp + fn); n. a. = not applicable; n. d. = not done.

**Table 3:** Evaluation of cutoff value for immunoreactive and immunonegative organs—automated organ selection.

Automated organ selection	n	fp	fn	tp	tn	gtp	gtn	Sensitivity	Specificity	Accuracy
Brain cutoff ≥ 0.09%										
Negative control	n. d.	n. d.	n. d.	n. d.	n. d.	n. d.	n. d.	n. d.	n. d.	n. d.
Training set	21	0	3	18	0	21	0	85.7%	n. a.	85.7%
Test set	12	0	1	10	1	11	1	90.9%	100.0%	91.7%
Validation set	13	0	2	11	0	13	0	84.6%	n. a	84.6%
Heart cutoff ≥ 0.13%										
Negative control	n. d.	n. d.	n. d.	n. d.	n. d.	n. d.	n. d.	n. d.	n. d.	n. d.
Training set	20	0	3	17	0	20	0	85.0%	n. a.	85.0%
Test set	12	0	1	10	1	11	1	90.9%	100.0%	91.7%
Validation set	15	0	2	11	2	13	2	84.6%	100.0%	86.7%
Lung cutoff ≥ 0.09%										
Negative control	n. d.	n. d.	n. d.	n. d.	n. d.	n. d.	n. d.	n. d.	n. d.	n. d.
Training set	21	0	3	15	3	18	3	83.3%	100.0%	85.7%
Test set	12	2	0	6	4	6	6	100.0%	66.7%	83.3%
Validation set	13	1	0	12	0	12	1	100.0%	0.0%	92.3%
Spleen cutoff ≥ 0.06%										
Negative control	n. d.	n. d.	n. d.	n. d.	n. d.	n. d.	n. d.	n. d.	n. d.	n. d.
Training set	20	1	7	12	0	19	1	63.2%	0.0%	60.0%
Test set	11	1	2	5	3	7	4	71.4%	75.0%	72.7%
Validation set	14	2	1	10	1	11	3	90.9%	33.3%	78.6%
Liver cutoff ≥ 0.12%										
Negative control	n. d.	n. d.	n. d.	n. d.	n. d.	n. d.	n. d.	n. d.	n. d.	n. d.
Training set	21	1	10	7	3	17	4	41.2%	75.0%	47.6%
Test set	11	0	4	3	4	7	4	42.9%	100.0%	63.6%
Validation set	13	1	4	6	2	10	3	60.0%	66.7%	61.5%
Kidney cutoff ≥ 0.06%										
Negative control	n. d.	n. d.	n. d.	n. d.	n. d.	n. d.	n. d.	n. d.	n. d.	n. d.
Training set	21	0	2	19	0	21	0	90.5%	n. a.	90.5%
Test set	12	0	1	10	1	11	1	90.9%	100.0%	91.7%
Validation set	3	0	2	1	0	3	0	33.3%	n. a.	33.3%

% = percentage of immunoreactive area; n: animal number; fp: false positive; fn: false negative; tp: true positive; tn: true negative; gtp: ground truth positive (= semiquantitative score > 0); gtn: ground truth negative (= semiquantitative score = 0); sensitivity = tp/gtp; specificity = tn/gtn; accuracy = (tp + tn)/(tp + tn + fp + fn); n. a. = not applicable; n. d. = not done.

### Correlation of immunohistochemical data with RT-qPCR

A correlation analysis of immunohistochemical and RT-qPCR data was done for samples of brain, heart, lung, spleen, liver, and kidney (n = 94) from the animals of study 1 (Supplementary Fig. S7). For the automatically detected percentage of immunoreactive area and the RT-qPCR data r was 0.4678 (Pearson’s correlation analysis, p < 0.0001). For the sum of the semiquantitative scores for parenchymal and endothelial antigen and the RT-qPCR results r was 0.5638 (Spearman’s correlation analysis, p < 0.0001).

## Discussion

This study aimed to develop a method for automated quantification of AIV antigen in different organs to reduce the workload for the involved pathologists. Based on our experience, the manual semiquantitative scoring of AIV antigen^8^ in the organs chosen for this study takes on average approximately 30 min per animal. In this study, the manual pre-selection of organs with a rough outline took about 15 min per animal, with an additional time of about 2 min for the unsupervised automated antigen quantification. In contrast, the fully automated, unsupervised analysis took about 9 min per animal. For the (partly) automated analyses, quality control of the results requires a small amount of extra time, which was approximately 2 min per animal for this study. Nonetheless, the time needed for the involved pathologist is markedly reduced, as the digitization of slides and the starting of the automated analysis can be done by trained technical staff. However, the training of the classifiers and the selection of the thresholds requires some time. Hence, the method presented here might not be the method of choice for small studies with low numbers of animals. Although it was only applied to a limited sample size in this study, this method might be useful for the analysis of larger sample sizes, as the automated part of the slide analysis takes only a fraction of the time needed for manual scoring after successful classifier training.

For most samples, the automated organ selection worked quite well, as was evaluated in detail for the small image regions with the Jaccard similarity index and for the whole slide visually and through comparison with a rough manual organ outline. The post-processing removal of smaller classified fragments turned out to be an important step for improving the selected regions. For some organs, particularly the kidney and the heart, the classifier-based organ selection was performing notably worse on the slide sets not used for classifier training (i.e., the test or validation set) compared to most other organs. Classifiers are known to often be limited by variability between different slides^14,23^. Furthermore, on the analyzed slides, multiple organ samples were present due to the design of the original studies and due to maintaining an efficient preparation and staining workflow. We found that the distinction of organs with a similar texture most likely presents a challenge to these classifiers, especially on immunohistochemical slides only stained with hematoxylin and the chromogen. On these slides, the organ structure itself is relatively low in contrasting colors as opposed to slides stained with hematoxylin and eosin. The kidney samples were often falsely classified as *liver* and as *background*. On the slide with the kidney sample, there was also a sample of glandular stomach present, which has a similar, tubule-like texture. As other, non-analyzed organs on the slide were labeled with the *background* class during training, this is most likely often responsible for the misclassification of the kidney as *background*. Therefore, to improve future studies, we suggest that slides for automated organ detection should not contain additional tissues not included in the analysis. In addition, the relatively similar structure of the brain and heart as well as of liver and kidney, respectively, probably led to the misclassification of the one for the other organ sometimes. This was mostly the case for slide sets not used for classifier training. Regarding the liver and the kidney, the selected organ area was further reduced by the automated exclusion of annotation margins due to the false immunoreactivity of some samples. These findings demonstrate the limitations of the automated organ selection for this study and should be considered in sample processing for further studies.

Despite digitization with standardized settings, the brightness of the background often varied between different slides and organs. Therefore, a post-processing step of automated small-scale background exclusion was omitted and only background areas of a size of 1,000,000 µm^2^ or larger were excluded from the surrounding organ area. This might have led to a certain amount of underestimation of the true immunoreactive area, especially in organs with many small, air-filled spaces such as the lung. Nonetheless, variability of selected areas for quantitative analysis is to be expected to some degree, regardless of the post-processing. For example, the airways might be filled with a varying amount of edematous fluid or erythrocytes due to pathological processes or euthanasia- and/or preparation-related artifacts, thus influencing the measured area.

To reduce the time required for the analysis, only one threshold was established per organ for measuring the immunoreactive area. Therefore, variation in staining intensity between different studies or batches of slides could affect the results, as a balance between true immunoreactive signal and background signal from other tissues must be found that works as best as possible over multiple slides. In this case, the validation set had a rather low staining intensity for AEC, which tended to underestimate the true immunoreactive area. In addition, in some slides of all sets, erythrocytes showed a false positive signal of low intensity, making it impossible to set a lower threshold. Furthermore, the resolution of the threshold-based pixel classifiers was set to “moderate” to shorten the analysis time, which may also have influenced the results of the antigen quantification.

Emphasis should also be put on the slide quality, as artifacts such as air bubbles, tissue folds or dust can easily impair the digitization^24^ as well as influence the image analysis itself^14^ and may lead to a false positive signal, as the computer cannot discern these from a true positive signal with the same ease as the human eye. Therefore, it is necessary to critically assess the quantification results and ensure the quality of the automated analysis^24^.

Spearman’s correlation analysis was done for automated and manual organ selection as well as for the semiquantitative parenchymal score only and the sum of the parenchymal and endothelial scores. In general, the correlation coefficients for the parenchymal scores were slightly higher than for the sum of parenchymal and endothelial scores, likely due to the smaller area taken up by immunoreactive endothelial cells compared to immunoreactive parenchymal cells. Furthermore, correlation coefficients for the liver and kidney were sometimes higher in the manual organ selection, especially for the validation set. This was probably influenced by the limitations of the automated organ selection regarding these organs.

Especially for the heart, liver, and kidney, the correlation coefficients in some slide sets were considerably lower than for the rest of the organs and sets. This might be due to the relatively high presence of background signals or to organ-specific differences in scoring criteria.

To further assess the additional cutoff value for whole organ positivity defined in this study, a larger scale validation is necessary, as the slide sets in this study show no equal distribution of truly positive and negative samples for all organs. Nonetheless, such a cutoff can give a quick overview of the organ tropism of different AIVs.

A comparison of the chickens of study 3 infected with H5N1 HPAIV and inoculated IV or ON showed a tendency towards larger immunoreactive areas and marked involvement of multiple organs for the IV-infected animals. The ON-infected animals generally had a smaller immunoreactive area. Interestingly, the largest amount of immunoreactive area for these animals was found in the lung, suggesting a possible influence of the oculonasal infection route. To our knowledge, so far no major differences in histopathological lesions and antigen distribution between IV and intranasal infection routes have been described for H5N1 HPAIV strains in chickens and both inoculation routes caused a systemic distribution of viral antigen and lesions^25,26^. Nonetheless, a shorter mean time to death has been reported for the IV route of inoculation compared to the intranasal route of inoculation^25,27^.

As immunohistochemical examination of AIV is often used in addition to RT-qPCR, we conducted further correlation analyses to compare these methods. We found a significant positive correlation between viral loads in multiple organs of the ON-infected animals from study 1 as detected by RT-qPCR and the corresponding immunohistochemical data. Although some studies have failed to demonstrate a coherent relationship between immunohistochemical and RT-PCR data^28^, our results are consistent with several other studies that have found a positive correlation. For instance, in ducks infected with H5N1 HPAIV^29,30^ or chickens and ducks infected with H5N8 HPAIV^8^. It is important to note that both techniques provide unique and valuable information contributing to a better understanding of AIV. For a fast and quantitative assessment of the overall amount of viral genome in an organ, including the intravascular blood, RT-qPCR is the preferred method. On the other hand, for topographic assessment of the relationship of the virus antigen to specific cell types and its association with lesions, immunohistochemistry is the preferred method^11^.

In summary, the automated random trees classifiers correctly selected most of the organ areas, and a suitable correlation was achieved between the immunoreactive area per organ and a manual semiquantitative score. A functional workflow was created and successfully applied to slides from various experimental studies of avian influenza. A great advantage of the open-source software QuPath is its ability to create one coherent script to perform multiple, consecutive steps. This allows for an easy automated processing of multiple slides, thus providing an easy and fast method for assessment of AIV tissue tropism and reducing the workload for the involved scientists. In conclusion, we have explored a method for high-throughput analysis of immunohistochemically stained whole slide images. This method can be used as tool to characterize the tissue tropism of avian influenza viruses in future studies.

### Supplementary Information

Supplementary Information.

## Data Availability

The datasets generated during and/or analysed during the current study are available from the corresponding author on reasonable request.
