# Supplementary material for: Automated quantification of avian influenza virus antigen in different organs
Source: Sci Rep. 2024 Apr 16;14:8766. doi: 10.1038/s41598-024-59239-5 (PMC11021523; doi:10.1038/s41598-024-59239-5)
Supplement: Supplementary file 1 — Supplementary Information. [file 41598_2024_59239_MOESM1_ESM.pdf]

Automated quantification of avian influenza virus antigen in different organs

Maria Landmann[1], David Scheibner[2], Marcel Gischke[2], Elsayed M. Abdelwhab[2], Reiner Ulrich[1]

[1]Institute of Veterinary Pathology, Leipzig University, Leipzig, Germany; [2]Institute of Molecular Virology and Cell Biology, Friedrich-Loeffler-Institut, Greifswald-Insel Riems, Germany

Parameter	Setting
Sample detection	Automatic threshold-based detection with manual optimization<br>Minimal region size 0.3 mm^2
Coarse focus map settings	10x objective, sample size-dependent with manual optimization:<br>From 0 mm^2: center of gravity<br>From 20 mm^2: fixed number of points (n = 3)<br>From 100 mm^2: fixed number of points (n = 6)
Fine focus map settings	20x objective, sample size-dependent with manual optimization:<br>From 0 mm^2: center of gravity<br>From 2 mm^2: every nth row and column (n = 2)<br>From 8 mm^2: every nth row and column (n = 4)<br>From 50 mm^2: every nth row and column (n = 5)<br>From 100 mm^2: onion skin (density = 0.05, maximum number of points = 24, level = 3, margin = 1)
Objective	20x (numeric aperture 0.45)
Condenser aperture	0.79*
Single plane/z-stack	single plane
Flash intensity	670 %
Flash duration	3 µs
Pixel size of scanned slides	0.1725 x 0.1725

Supplementary Table S1. Detailed settings for AxioScan 7. *chosen due to artifacts at lower aperture

Name	Values
Hematoxylin	0.8342; 0.5312; 0.14812
DAB*	0.17378; 0.55713; 0.81204
Background	255; 255; 255

* names are pre-set by QuPath

Supplementary Table S2. Settings for stain vectors.
DAB was adapted for AEC staining

Name	Values
sigmaMicrons	5.0
spacingMicrons	75.0
maxIterations	10
regularization	0.01
adaptRegularization	true
useDeconvolved	true

Supplementary Table S3. Settings for “SLIC superpixel segmentation” plugin

Name	Values
pixelSizeMicrons	1.0
region	ROI
tileSizeMicrons	25.0
colorOD	true
colorStain1	true
colorStain2	true
colorStain3	true
colorRed	true
colorGreen	true
colorBlue	true
colorHue	true
colorSaturation	true
colorBrightness	true
doMean	true
doStdDev	true
doMinMax	true
doMedian	true
doHaralick	true
haralickDistance	1
haralickBins	32

Supplementary Table S4. Settings for calculation of intensity features

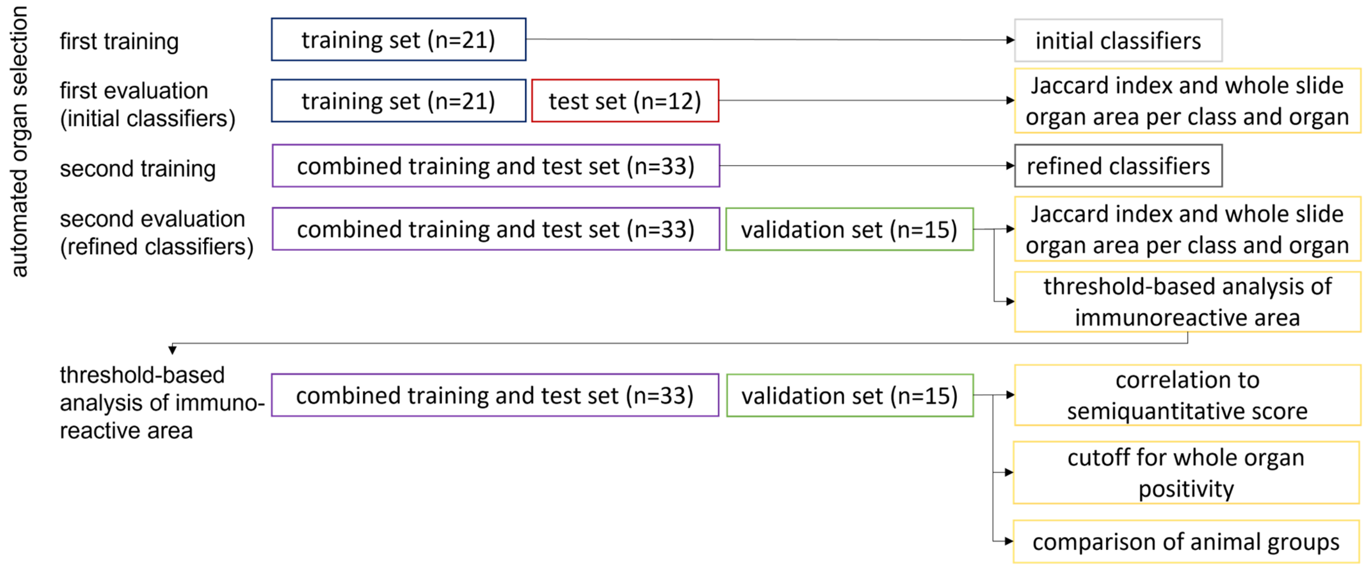

Supplementary Figure S1. Overview of training and evaluation process

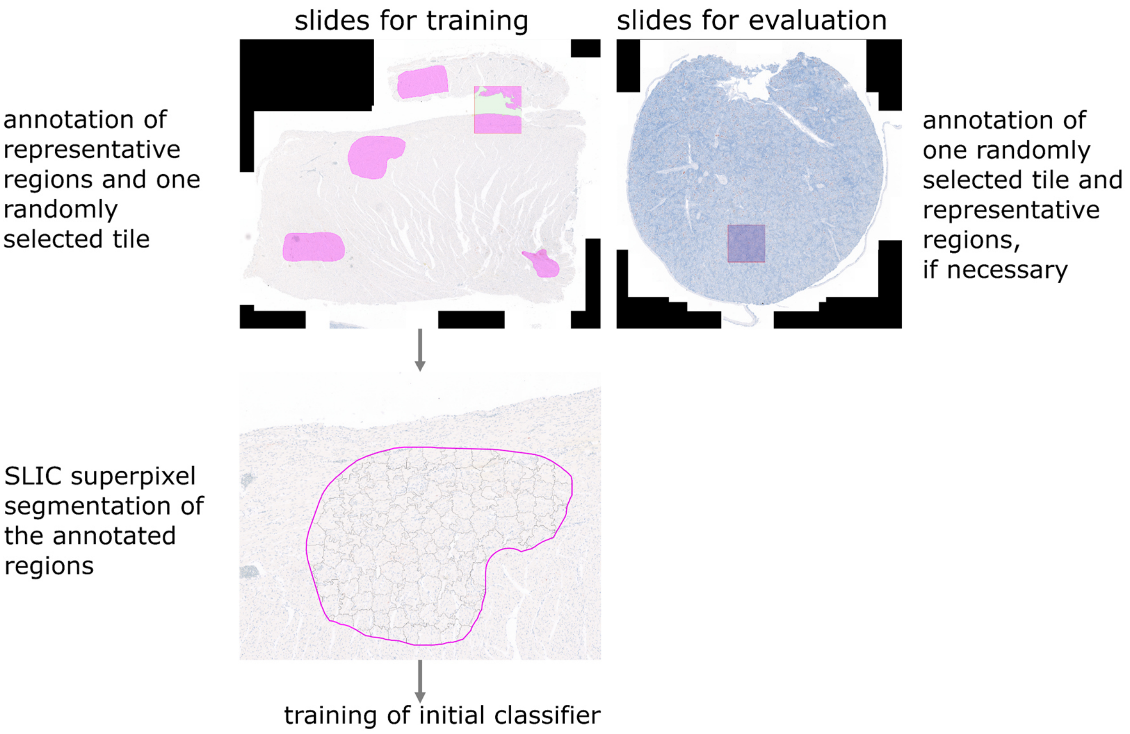

Supplementary Figure S2. Image analysis workflow – training of the classifiers

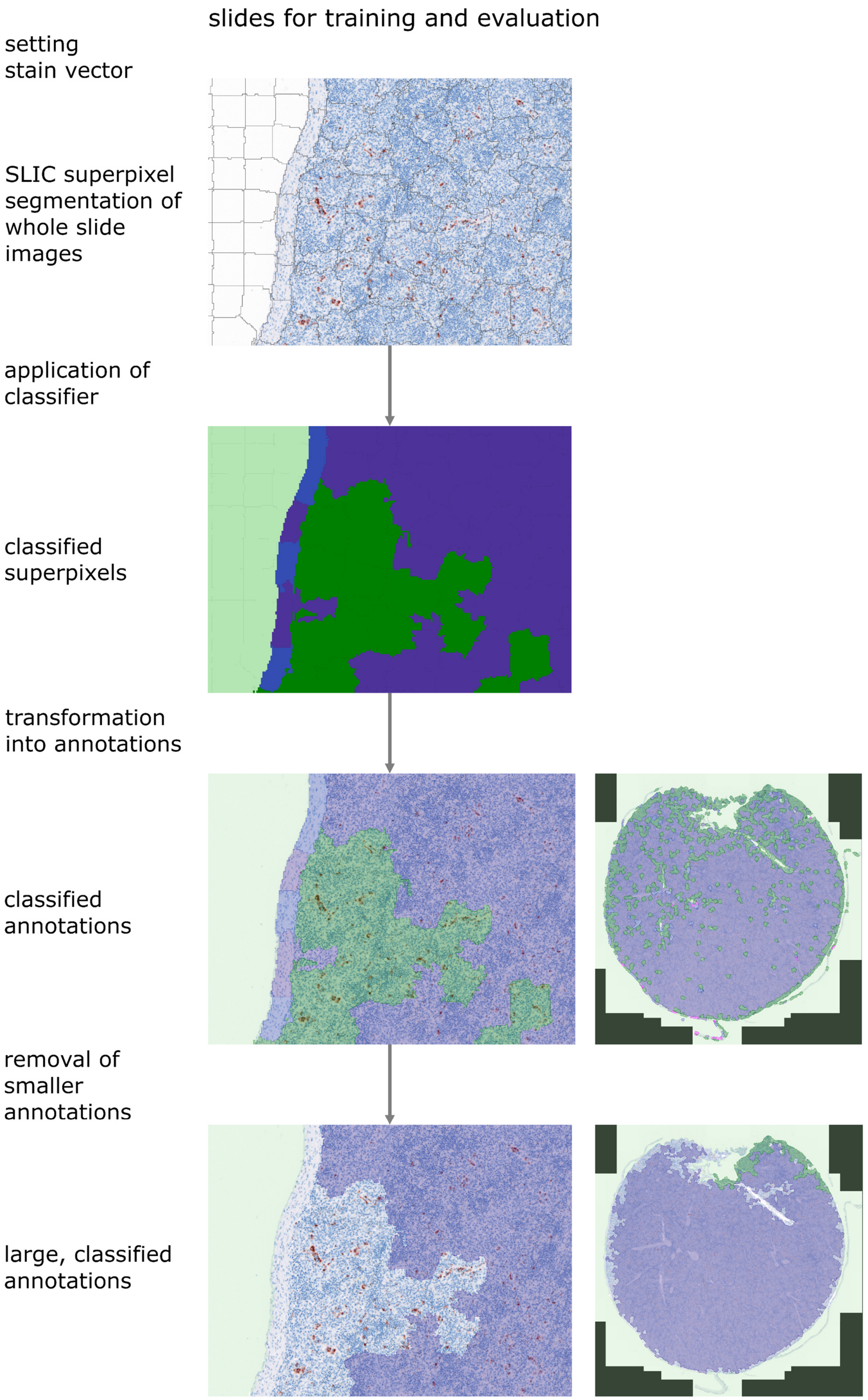

Supplementary Figure S3. Image analysis workflow – application of the classifiers

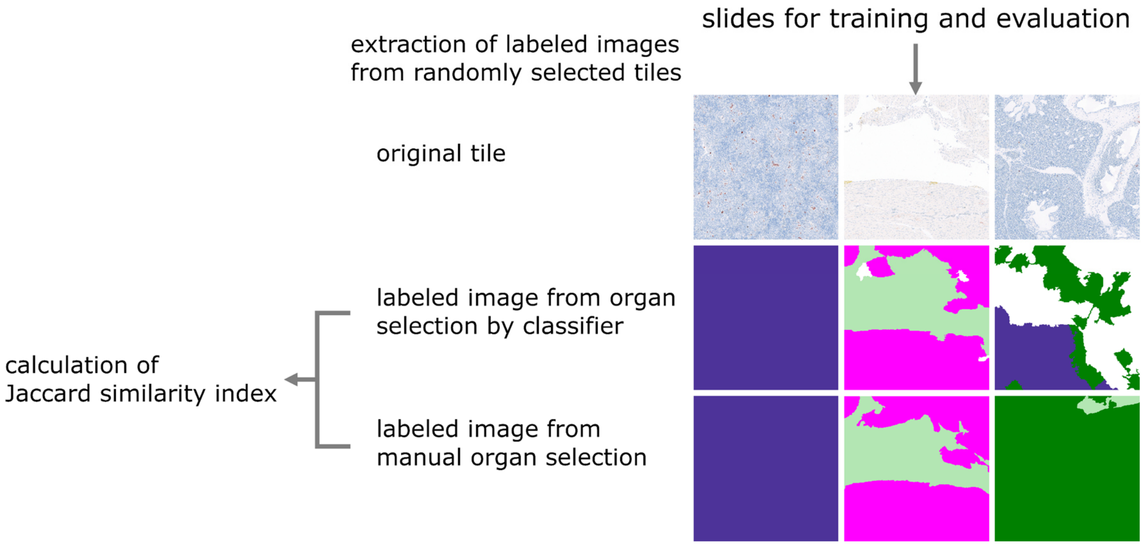

Supplementary Figure S4. Image analysis workflow – labeled images and Jaccard similarity index

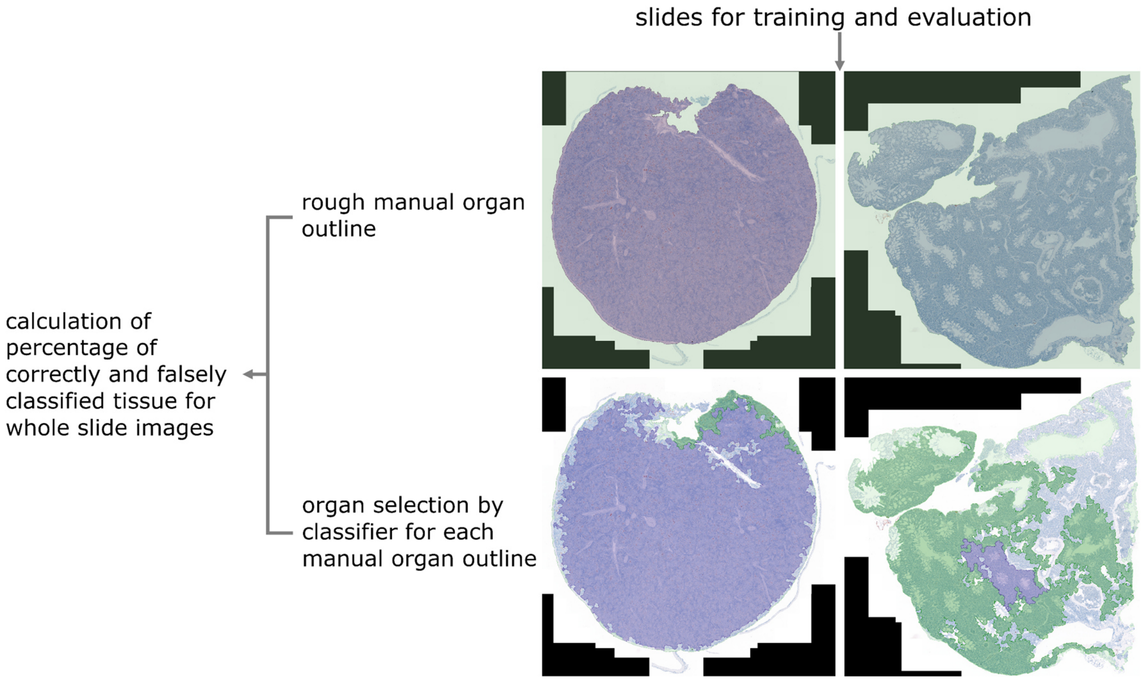

Supplementary Figure S5. Image analysis workflow – comparison to manual organ outline

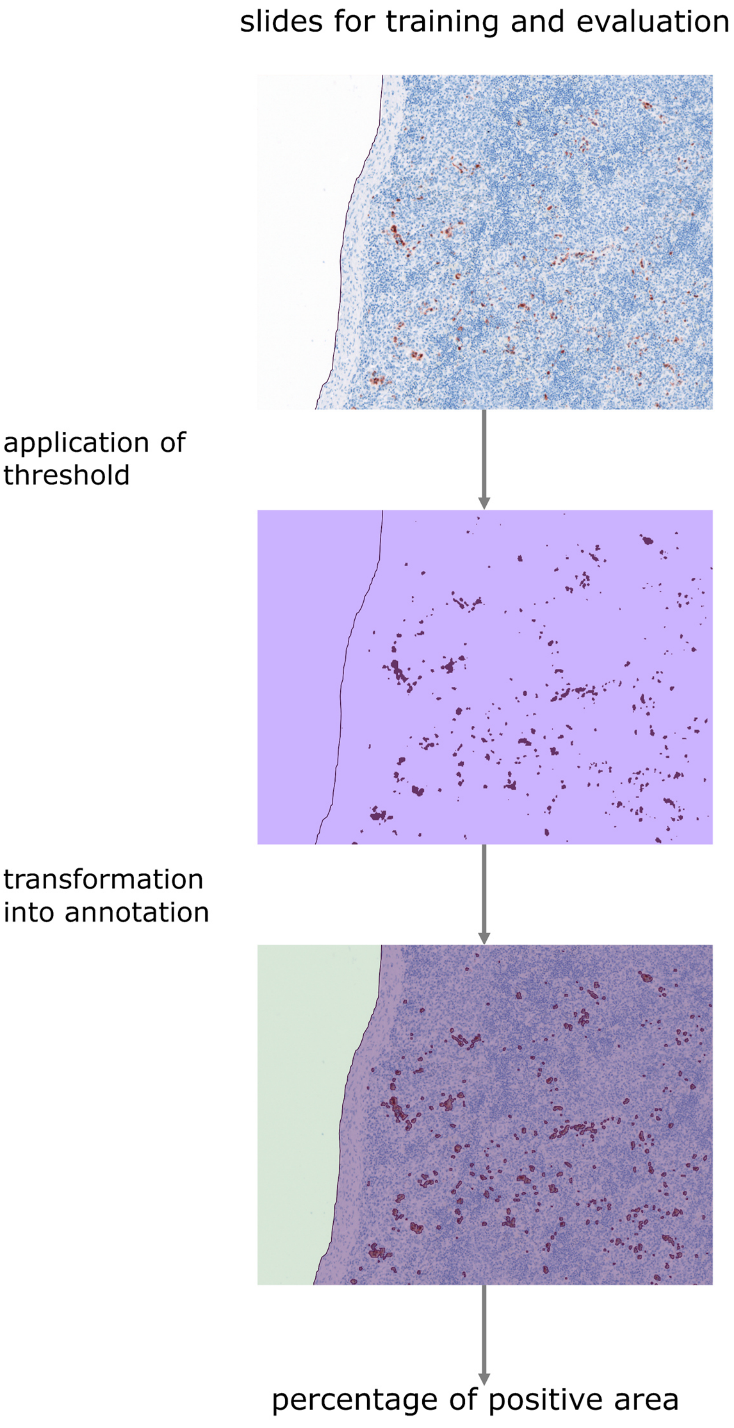

Supplementary Figure S6. Image analysis workflow – threshold-based quantification of immunoreactive area

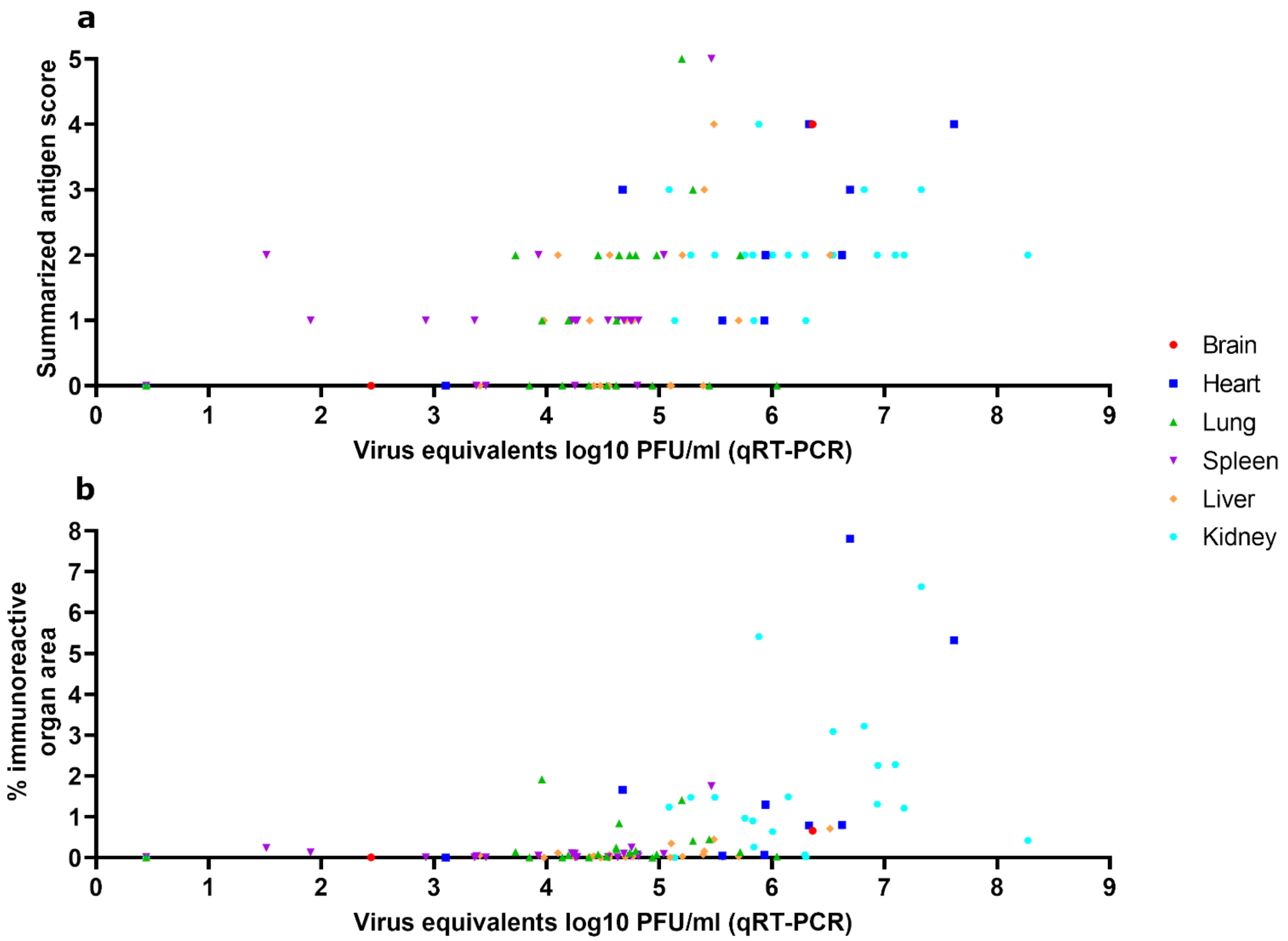

Supplementary Figure S7. Viral RNA loads (in virus equivalents log10 PFU/ml) for different organ samples (n = 94) from chickens of study 1 and **a:** sum of semiquantitative scores for parenchymal and endothelial antigen or **b:** threshold-based percentage of immunoreactive organ area measured in the automated organ selection. For both, there was a significant positive correlation ($p < 0.0001$) with $r = 0.5638$ for the semiquantitative score (Spearman's correlation analysis) and $r = 0.4678$ (Pearson's correlation analysis) for the percentage of immunoreactive organ area.
